# Supplementary material for: The association between dietary inflammatory index and non-alcoholic fatty liver disease: A systematic review and meta-analysis
Source: PLoS One. 2026 Mar 20;21(3):e0345297. doi: 10.1371/journal.pone.0345297 (PMC13004392; doi:10.1371/journal.pone.0345297)
Supplement: S2 Table — (DOCX) [file pone.0345297.s002.docx]

S2 Table Literature search strategy

1.Pubmed-5702

((((dietary inflammatory index) OR (dietary inflammatory score)) OR (DII)) OR (dietary inflammatory)) AND (("Non-alcoholic Fatty Liver Disease"[Mesh]) OR ((((((((((((((((Fatty Liver, Nonalcoholic) OR (Fatty Livers, Nonalcoholic)) OR (Liver, Nonalcoholic Fatty)) OR (Livers, Nonalcoholic Fatty)) OR (Nonalcoholic Fatty Liver)) OR (Nonalcoholic Fatty Livers)) OR (NAFLD)) OR (Nonalcoholic Fatty Liver Disease)) OR (Nonalcoholic Steatohepatitis)) OR (Nonalcoholic Steatohepatitides)) OR (Steatohepatitides, Nonalcoholic)) OR (Steatohepatitis, Nonalcoholic)) OR (fatty liver)) OR (MASLD)) OR (metabolic dysfunction-associated steatohepatitis)) OR (MASH)))

2.Embase-63


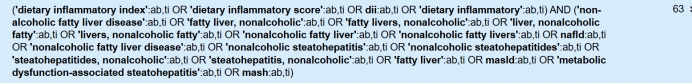


1. Cochrane-79


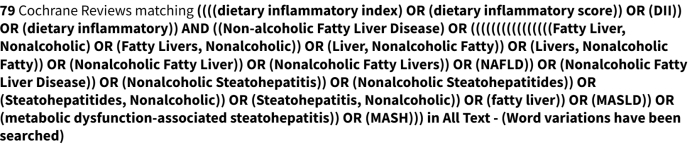


1. Web of science-37

((((dietary inflammatory index) OR (dietary inflammatory score)) OR (DII)) OR (dietary inflammatory)) AND ((Non-alcoholic Fatty Liver Disease) OR ((((((((((((((((Fatty Liver, Nonalcoholic) OR (Fatty Livers, Nonalcoholic)) OR (Liver, Nonalcoholic Fatty)) OR (Livers, Nonalcoholic Fatty)) OR (Nonalcoholic Fatty Liver)) OR (Nonalcoholic Fatty Livers)) OR (NAFLD)) OR (Nonalcoholic Fatty Liver Disease)) OR (Nonalcoholic Steatohepatitis)) OR (Nonalcoholic Steatohepatitides)) OR (Steatohepatitides, Nonalcoholic)) OR (Steatohepatitis, Nonalcoholic)) OR (fatty liver)) OR (MASLD)) OR (metabolic dysfunction-associated steatohepatitis)) OR (MASH)))(Title)

1. Scopus-2900

TITLE-ABS-KEY ( ( ( ( ( dietary inflammatory index ) OR ( dietary inflammatory score ) ) OR ( DII ) ) OR ( dietary inflammatory ) ) AND ( ( Non-alcoholic Fatty Liver Disease ) OR ( ( ( ( ( ( ( ( ( ( ( ( ( ( ( ( Fatty Liver , Nonalcoholic ) OR ( Fatty Livers , Nonalcoholic ) ) OR ( Liver , Nonalcoholic Fatty ) ) OR ( Livers , Nonalcoholic Fatty ) ) OR ( Nonalcoholic Fatty Liver ) ) OR ( Nonalcoholic Fatty Livers ) ) OR ( NAFLD ) ) OR ( Nonalcoholic Fatty Liver Disease ) ) OR ( Nonalcoholic Steatohepatitis ) ) OR ( Nonalcoholic Steatohepatitides ) ) OR ( Steatohepatitides , Nonalcoholic ) ) OR ( Steatohepatitis , Nonalcoholic ) ) OR ( fatty liver ) ) OR ( MASLD ) ) OR ( metabolic dysfunction-associated steatohepatitis ) ) OR ( MASH ) ) ) )
