# Supplementary material for: The association between dietary inflammatory index and non-alcoholic fatty liver disease: A systematic review and meta-analysis
Source: PLoS One. 2026 Mar 20;21(3):e0345297. doi: 10.1371/journal.pone.0345297 (PMC13004392; doi:10.1371/journal.pone.0345297)
Supplement: S3 Table — (DOCX) [file pone.0345297.s003.docx]

S3 Table Quality evaluation of the eligible studies with Newcastle–Ottawa scale

| Study | Selection | | | | Comparability | | Outcome | | |
| --- | --- | --- | --- | --- | --- | --- | --- | --- | --- |
|  | Representative-ness | Selection of  non-exposed | Ascertainment  of exposure | Outcome not present at start | Comparability on most important factors | Comparability on other risk factors | Assessment of outcome | Long enough follow-up (median≥1 year) | Adequacy  (completeness) of follow-up |
| Vahid2018 | * | * | * | * | - | * | * | * | * |
| Mazidi2018 | * | * | * | * | - | - | * | * | * |
| Ramírez-Vélez2021 | * | * | * | * | - | - | * | * | * |
| Li2022 | * | * | * | * | - | - | * | * | * |
| Heidari2022 | * | * | * | * | - | - | * | * | * |
| Tian2022 | * | * | * | * | - | - | * | * | * |
| Li2023 | * | * | * | * | - | - | * | * | * |
| Zhang2023 | * | * | * | * | - | - | * | * | * |
| Shi2023 | * | * | * | * | * | - | * | * | * |
| Petermann-Rocha2023 | * | * | * | * | - | - | * | * | * |
| Valibeygi2023 | * | * | * | * | - | - | * | * | * |
| Soltanieh2023 | * | * | * | * | - | * | * | * | * |
| Li2024 | * | * | * | * | - | - | * | * | * |
| Xu2024 | * | * | * | * | - | - | * | * | * |
| Yan2024 | * | * | * | * | - | - | * | * | * |
| Motamedi2024 | * | * | * | * | - | - | * | * | * |
| Hu2024 | * | * | * | * | - | - | * | * | * |
| Doustmohammadian 2024 | * | * | * | * | - | - | * | * | * |

*indicates criterion met; - indicates significant of criterion not met
