# Supplementary figures and images for: The association between dietary inflammatory index and non-alcoholic fatty liver disease: A systematic review and meta-analysis
Source: PLoS One. 2026 Mar 20;21(3):e0345297. doi: 10.1371/journal.pone.0345297 (PMC13004392; doi:10.1371/journal.pone.0345297)

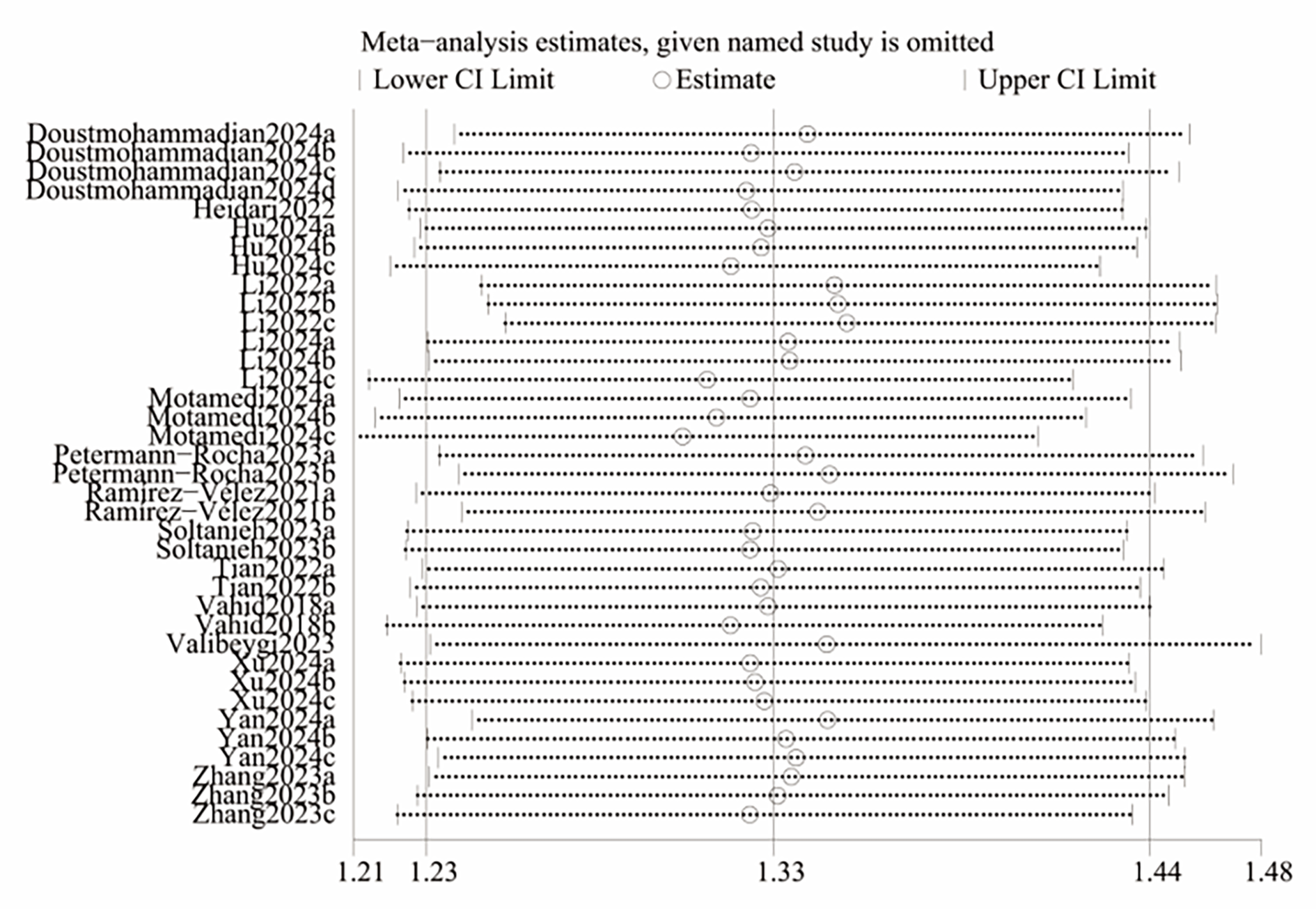

Supplement: S1 Fig — (TIF) [file pone.0345297.s004.tif]

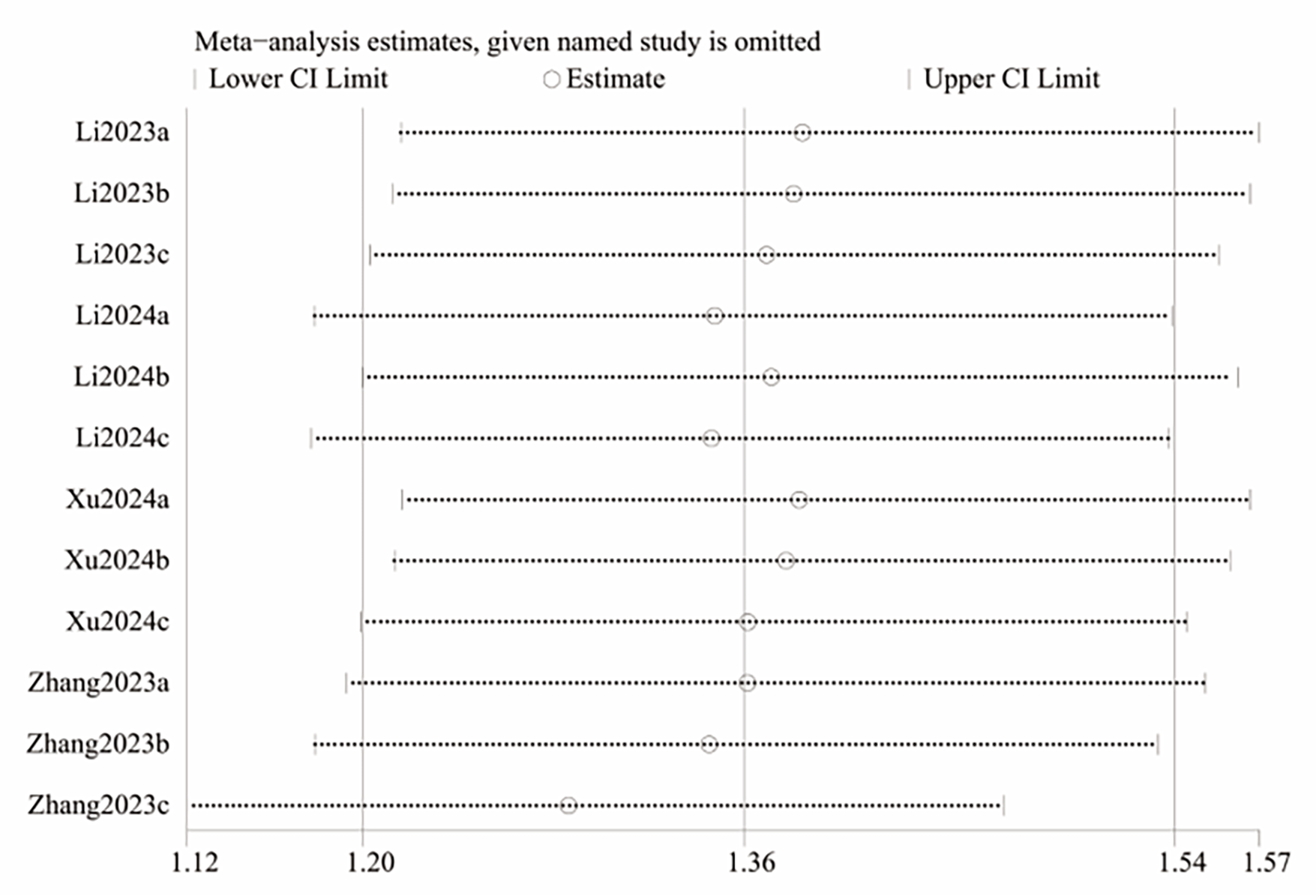

Supplement: S2 Fig — (TIF) [file pone.0345297.s005.tif]

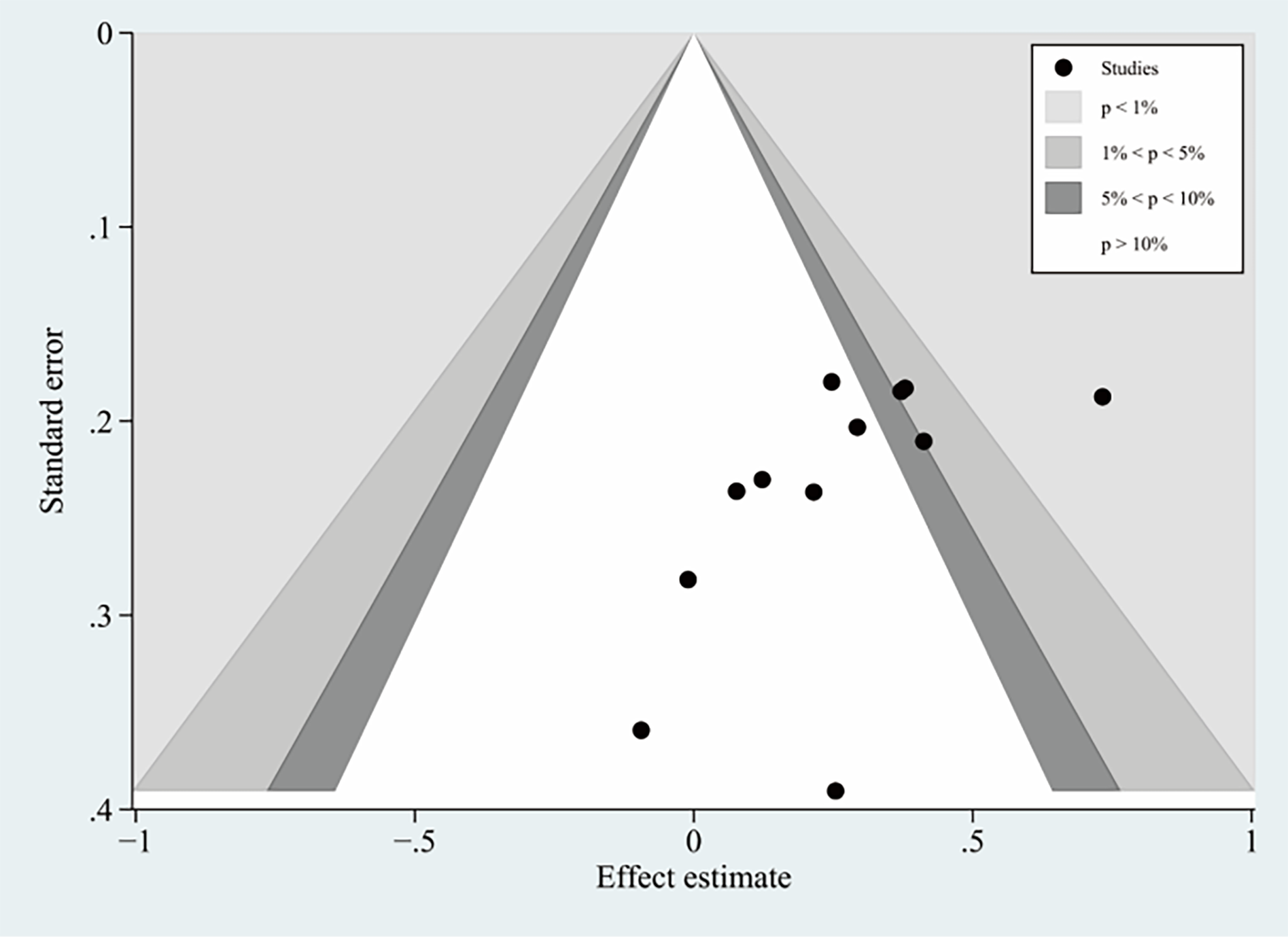

Supplement: S3 Fig — (TIF) [file pone.0345297.s006.tif]
