# Supplementary material for: The association between dietary inflammatory index and non-alcoholic fatty liver disease: A systematic review and meta-analysis
Source: PLoS One. 2026 Mar 20;21(3):e0345297. doi: 10.1371/journal.pone.0345297 (PMC13004392; doi:10.1371/journal.pone.0345297)
Supplement: Attachment — Submitted filename: response.doc [file pone.0345297.s007.doc]

Subject: Clarification on Distinctiveness and Innovation of Our Submitted Manuscript

Dear Editor,

We sincerely appreciate your review of our manuscript titled “The Association Between Dietary Inflammatory Index and Non-Alcoholic Fatty Liver Disease: A Systematic Review and Meta-Analysis” and your valuable feedback regarding its similarity to the systematic review and meta-analysis published by Amirkalali et al. in 2025. We have carefully examined their work and fully understand your concern about possible overlap.

Here, we would like to elaborate on the key differences between the two papers and highlight the unique contributions and innovations of our study.

I. Key Differences Between the Two Studies

1. Scope and Depth of the Research

Amirkalali et al. (2025): Their study primarily focused on the association between the Dietary Inflammatory Index (DII) and the risk of NAFLD development. Based on 11 observational studies (9 cross-sectional and 2 cohort), they addressed the question: “Does a pro-inflammatory diet increase the likelihood of developing NAFLD?”

Our Study: We extended the scope from disease incidence to disease progression. For the first time in a systematic review and meta-analysis, we not only examined the association between DII and NAFLD occurrence but also specifically quantified the relationship between DII and the risk of progression to liver fibrosis in NAFLD patients. This addresses a more clinically relevant question: “Does a pro-inflammatory diet not only contribute to fatty liver but also accelerate its progression to more severe liver injury?” Thus, our study covers a broader spectrum of disease outcomes with deeper clinical implications.

2. Study Endpoints and Included Research

Amirkalali et al. (2025): The primary outcome was NAFLD prevalence/incidence, focusing on observational studies linking DII to NAFLD.

Our Study: We established dual primary outcomes: (1) NAFLD and (2) liver fibrosis. During the literature search and screening process, we specifically included studies that reported fibrosis-related indicators (such as FIB-4, APRI, liver stiffness measurement, etc.). Our final analysis incorporated 18 studies with 262,468 participants—a larger and more updated evidence base—and for the first time, we extracted data from 12 comparison groups to report the pooled effect size for DII and fibrosis risk (OR = 1.36). This analysis of fibrosis represents a novel contribution not addressed in previous reviews.

3. Analytical Dimensions and Novel Findings

Amirkalali et al. (2025): They conducted subgroup analyses exploring the influence of factors such as BMI, geographical region, and diagnostic methods on the DII–NAFLD association.

Our Study: In addition to validating the heterogeneity of the DII–NAFLD association across regions and diagnostic criteria, we pioneered subgroup analyses specifically for the DII–fibrosis relationship. For instance, we found that the association was more stable in larger sample sizes (>5,000 participants) and that the Fatty Liver Index (FLI) showed particular utility in identifying fibrosis risk. These insights offer new directions for future research design and clinical tool selection.

4. Terminology and Clinical Context

We explicitly acknowledged the recent transition in terminology from NAFLD to MASLD (Metabolic Dysfunction-Associated Steatotic Liver Disease) in our discussion. We retained the term “NAFLD” to maintain consistency with the historical studies included in our analysis, ensuring accurate data interpretation while demonstrating our awareness of the evolving field.

II. Innovations of Our Study

First Meta-Analysis on DII and Liver Fibrosis: Our study is the first systematic review and meta-analysis to quantitatively demonstrate that higher DII scores are significantly associated with an increased risk of liver fibrosis in NAFLD patients. This provides the highest level of synthesized evidence on the role of dietary inflammation in NAFLD progression, filling a critical gap in the existing literature.

Expanded Clinical Relevance of DII in Hepatology: Beyond reinforcing DII as a risk marker for NAFLD, our findings suggest that DII could serve as a simple dietary assessment tool to identify NAFLD patients at higher risk of disease progression. This may facilitate risk stratification and early, intensive intervention in clinical practice.

Comprehensive and Updated Evidence Synthesis: By including more recent studies and a larger sample size, we have updated the evidence on the DII–NAFLD relationship. Furthermore, by incorporating fibrosis as an endpoint, we provide a more comprehensive and in-depth overview of the association between dietary inflammation and the spectrum of fatty liver disease.

In-Depth Exploration of Heterogeneity: Our subgroup analysis for fibrosis outcomes revealed new sources of heterogeneity (e.g., sample size), offering methodological insights that can inform the design of future studies in this area.

Summary

In essence, while the study by Amirkalali et al. effectively answers the question, “Is dietary inflammation related to the development of NAFLD?” our research takes a significant step forward by addressing, for the first time via meta-analysis, the more prognostically relevant question: “Is dietary inflammation associated with disease progression?” Although there is some overlap in the topics, the focus, endpoints, analytical dimensions, and core conclusions of the two studies are fundamentally distinct. Our work represents an important extension and deepening of the existing evidence.

We firmly believe that the new evidence presented in our study on the association between DII and liver fibrosis holds unique value for the fields of hepatology and nutrition, as well as for clinical practice. Thank you once again for your careful review. We hope this clarification assists in your evaluation.

Best regards,

Jingjing Lin, Mengna Huang, Lina Shen

December 10, 2025
