# Supplementary material for: The association between dietary inflammatory index and non-alcoholic fatty liver disease: A systematic review and meta-analysis
Source: PLoS One. 2026 Mar 20;21(3):e0345297. doi: 10.1371/journal.pone.0345297 (PMC13004392; doi:10.1371/journal.pone.0345297)
Supplement: Attachment — Submitted filename: response_auresp_1.doc [file pone.0345297.s008.doc]

**Dear Editors and Reviewers:**

Thank you for your letter and for the reviewers’ comments concerning our manuscript entitled “**The Association Between Dietary Inflammatory Index and Non-Alcoholic Fatty Liver Disease: A Systematic Review and Meta-Analysis**”(PONE-D-25-65585). Those comments are all valuable and very helpful for revising and improving our paper, as well as the important guiding significance to our researches. We have studied comments carefully and have made correction which we hope meet with approval. Revised portion are marked in red in the paper. The main corrections in the paper and the responds to the editor and reviewer’s comments are as flowing:

Responds to the editor’s comments:

**Editor #：**

1. **Comment.** Data inconsistencies exist between the abstract, flowchart, and Table 1 regarding study numbers and participant totals that need verification.

**Response:** Regarding the issue of data inconsistencies, we have thoroughly verified the number of studies and total participants in the abstract, flowchart, and Table 1. The discrepancies were mainly due to inconsistent counting of different comparison groups within the same study. We have now standardized this: the abstract and flowchart clearly report the number of independent studies (18), while Table 1 details all comparison groups (43) and their corresponding cumulative sample size (262,468 participants).

2. **Comment.** The substantial heterogeneity (I²=74%) is inadequately explained despite subgroup analyses

**Response:** Regarding insufficient explanation of heterogeneity, we have added a paragraph in the discussion section systematically outlining potential sources of heterogeneity: focusing on differences in DII calculation methods (such as inconsistent parameters in FFQ versus 24-hour recalls), baseline characteristics of populations (such as age and metabolic comorbidities), and regional dietary pattern differences affecting pooled effect sizes. We have also addressed this issue in the limitations section, reminding readers to consider the impact of heterogeneity when interpreting the results.

3. **Comment.** The Discussion insufficiently addresses how variations in DII calculation methods and dietary assessment tools may have contributed.

**Response:** Regarding insufficient depth in the discussion, we have rewritten the paragraph on "methodological limitations of the DII" to emphasize the impact of different dietary assessment tools and calculation methods on DII accuracy.

4. **Comment.** Publication bias was detected for NAFLD (Egger's p=0.014), warranting more cautious interpretation rather than the current definitive conclusions.

**Response:** Regarding the rigor of the conclusions, we have revised the statements to "evidence suggests a possible association" and noted that "future studies are needed to adjust for methodological differences to confirm the strength of the association," aligning the conclusions with the strength of the evidence.

5. **Comment.** The frailty analysis based on only two comparison groups from a single study provides insufficient evidence and should be removed or clearly designated as exploratory.

**Response:** Regarding the frailty analysis, we fully agree with your assessment. In the results section, we clearly stated: "The frailty analysis included only 2 comparison groups with a limited sample size, so the results should be interpreted with caution." We also suggested in the discussion limitations that "future studies are needed to verify the association between DII and frailty," avoiding overinterpretation of the current data.

6. **Comment.** The MASLD nomenclature discussion is overly lengthy and could be condensed.

**Response:** Regarding the length of the MASLD terminology discussion, we condensed the original background explanation to a paragraph under 100 words, retaining only the key information: "Although the 2023 consensus recommends using the MASLD term, the studies included in this research all used the NAFLD definition. To maintain consistency with the original research, this study continues to use the NAFLD terminology; this difference does not affect the analysis of fatty liver disease itself." This saves space for deeper methodological discussion.

7. **Comment.** Language errors appear throughout, including "TGrowing" in the Introduction and inconsistent reference formatting.

**Response:** Regarding language errors, we conducted a full manuscript proofread: correcting the spelling error in the introduction from "TGrowing" to "The growing," standardizing reference formatting (e.g., journal abbreviations, author formats), and refining awkward sentences. Additionally, we hired a native editor to polish the entire manuscript to ensure proper and fluent expression.

**Reviewer #1:**

1. **Comment.** high heterogeneity (I²=74% for NAFLD) persists overall, potentially due to DII calculation variations (e.g., FFQ vs. 24h recall, parameter differences), population baselines, or confounders.

**Response:** Regarding the high heterogeneity issue you pointed out, we have added a paragraph in the discussion section to systematically explain the potential sources of heterogeneity: we focused on analyzing the impact of differences in DII calculation methods (such as inconsistencies in parameters between FFQ and 24-hour recalls), population baseline characteristics (such as age and metabolic comorbidities), and geographic dietary patterns on the pooled effect size; meanwhile, we have addressed this issue in the limitations section, reminding readers to consider the influence of heterogeneity when interpreting the results.

2. **Comment.** Publication bias (Egger's p=0.014 for NAFLD) raises concerns, though mitigated.

**Response:** Concerning the issue about publication bias, we have reinforced the relevant statements in the limitations part of the discussion: clearly indicating that the Egger test (p=0.014) suggests the presence of potential publication bias, but the effect size remains significant after correction using the trim-and-fill method (OR=1.205), indicating that the main conclusions are robust; additionally, we have added a note stating that "the bias may stem from unpublished studies with small sample sizes showing negative results."

3. **Comment.** Frailty analysis is underpowered (limited studies), limiting robustness.

**Response:** Regarding the insufficiency of frailty analysis, we fully agree with your observation. In the results section, we explicitly stated: "The frailty analysis included only 2 comparison groups, with a limited sample size, so the results should be interpreted with caution"; at the same time, in the discussion of limitations, we also suggested that "future research is needed to further verify the association between DII and frailty," thereby avoiding overinterpreting the current data.

4. **Comment.** Statistical analysis is appropriate and rigorous. The authors state data supporting findings are included in the article. The manuscript is intelligible and in standard English. Structure follows PRISMA and language is clear, professional.

Response: We express our gratitude for the affirmation of the statistical analysis methods and the recognition of the language and structure.

**Reviewer #2:**

**1.Comment.** The Introduction section should contain more information regarding new nomenclature (MASLD).

**Response:** Regarding the suggestion to supplement the introduction with background on the new nomenclature (MASLD), we have added a paragraph in the introduction detailing the origin of the term metabolic dysfunction-associated steatotic liver disease (MASLD), its key differences from NAFLD in diagnostic criteria, and explaining why this study continues to use the term NAFLD based on the literature inclusion time frame and consistency considerations. This helps readers more comprehensively understand the evolution of the terminology and its potential implications for the context of this study.

2. **Comment.** The abbreviations, when first introduced, should be used consistently thereafter throughout the text.

**Response:** Regarding the suggestion on consistency in abbreviation use, we have systematically checked and revised the entire text to ensure that all abbreviations are clearly defined in the form of "full term (abbreviation)" upon first appearance (e.g., metabolic dysfunction-associated steatotic liver disease (MASLD)) and uniformly use the abbreviation in subsequent text.

We tried our best to improve the manuscript and made some changes in the manuscript. These changes will not influence the content and framework of the paper. And here we did not list the changes but marked in red in revised paper.

We appreciate for Editors/Reviewers’ warm work earnestly, and hope that the correction will meet with approval.

Once again, thank you very much for your comments and suggestions.
